# Feedback modalities in human-cobot collaboration: experimental evaluation of performance, user experience, and physiological responses

**DOI:** 10.3389/frobt.2026.1836165

**Published:** 2026-07-30

**Authors:** Amir Biton, Yuval Cohen, Shraga Shoval

**Affiliations:** 1 Department of Industrial Engineering and Management, Ariel University, Ari'el, Israel; 2 Department of Engineering, Afeka College of Engineering, Tel Aviv-Yafo, Israel

**Keywords:** assistive interface, collaborative robots, feedback modality, heart rate variability, human factors, human-robot collaboration, physiological measures

## Abstract

Collaborative robots (cobots) are increasingly deployed in industrial as well as non-industrial domains to support human-centered operation. While physical safety and task efficiency have received considerable attention, less is known about how feedback modality influences operator experience and physiological responses under different collaboration demands. This study examines the effects of feedback modalities in two human–cobot collaboration scenarios representing distinct coordination structures: a low-collaboration turn-taking task and a high-collaboration synchronous shared-control task. Across these scenarios, auditory, visual, and combined auditory–visual feedback modalities were evaluated, and an additional projected assistive interface (PAI) condition, designed specifically for the given scenario, was introduced in the high-collaboration task. Outcomes included task completion time, subjective user assessments of interaction quality and comfort, and physiological responses based on heart rate variability (HRV) indicators reflecting autonomic activity. The results indicate that feedback modality significantly influences performance, subjective experience, and physiological activation within each collaboration scenario. In the low-collaboration task, the no-feedback condition yielded the shortest completion times but was associated with lower perceived interaction quality and descriptively higher autonomic activation. In contrast, multimodal feedback improved perceived comfort and task understanding while slightly increasing completion time. In the high-collaboration task, the examined PAI guidance condition was associated with the shortest task completion times and the highest subjective ratings, while also being accompanied by increased sympathetic-dominant physiological activation. These findings indicate a design trade-off in which richer guidance can enhance coordination efficiency but may be associated with increased cognitive and physiological demands. Importantly, the results suggest that physiological activation should be interpreted in relation to task demands and performance outcomes, rather than as a direct indicator of detrimental stress. More broadly, the study suggests that collaboration configuration and feedback modality should be co-designed as interdependent parameters within the specific coordination demands of collaborative human–robot interaction tasks. Although the PAI was designed for this specific scenario, future research may examine whether similarly tailored interfaces can support positive user evaluations in other complex collaborative tasks. By integrating subjective and physiological measures, this work provides evidence-informed guidance for designing feedback strategies that support efficient, safe, and sustainable human–cobot collaboration.

## 1 Introduction

Collaborative robots (cobots) are increasingly deployed to support flexible and human-centered production. Unlike traditional industrial robots that operate in segregated environments, cobots are designed to share workspaces with humans and coordinate missions in close physical and temporal proximity (Villani et al., 2018; Bauer, 2016). While advances in sensing, control, and safety engineering have significantly reduced physical risks (Haddadin et al., 2017), effective deployment of cobots requires attention not only to physical safety but also to how operators perceive, interpret, and coordinate with robotic partners during interaction. From an ergonomics perspective, the design of interaction cues and feedback mechanisms plays a central role in shaping human performance, user experience, and workload in collaborative work systems.

Recent research in human–robot collaboration has increasingly emphasized the importance of cognitive ergonomics and operator-centered design. Beyond objective safety and efficiency, factors such as perceived safety, workload, and interaction transparency can influence operator behavior, decision-making, and long-term acceptance of collaborative technologies (Ajoudani et al., 2018; Hancock et al., 2011; Lasota et al., 2017). Empirical studies in industrially motivated assembly contexts further demonstrate that collaboration conditions and interaction mechanisms can significantly affect completion time, error rates, workload, and acceptability (Fournier et al., 2024; Su et al., 2024; Lu et al., 2024). These findings highlight the need for interface design approaches that explicitly account for operator-centered outcomes when configuring collaborative robot workstations.

To better understand operator state during human–robot interaction, recent studies have increasingly incorporated physiological measurements alongside subjective assessments. Physiological indicators such as heart rate variability (HRV) provide objective insights into autonomic regulation and can complement self-reported measures by capturing aspects of operator state that may not be consciously perceived or easily articulated (Charles and Nixon, 2019; Dehais et al., 2020). In human–robot collaboration, physiological sensing has therefore been proposed as a promising tool for evaluating workload, stress-related responses, and sustainable operator performance during collaborative tasks (Gualtieri et al., 2021; Hopko et al., 2023).

Despite these advances, relatively limited empirical evidence exists on how feedback modality design influences both operator experience and physiological responses during human–robot collaboration. Existing work often focuses on isolated interaction mechanisms or narrowly defined experimental tasks, limiting the ability to derive practical design implications for collaborative workstations. In particular, little is known about how different feedback modalities affect operator performance, subjective experience, and physiological activation across interaction contexts that impose different coordination demands.

To address this gap, the present study investigates feedback modality effects in two human–cobot interaction scenarios representing different coordination structures. In the first scenario, human and robot actions follow a sequential turn-taking pattern representing a low level of collaboration, whereas the second scenario requires continuous synchronous coordination between the operator and the cobot, representing a high level of coordination. Within each scenario, multiple feedback modalities were evaluated, including auditory, visual, multimodal auditory–visual cues, and in the synchronous task a spatial guidance condition implemented through projected assistive interface-style cues (PAI) designed to assist the user in the specific complex task.

By combining task performance measures, subjective self-report assessments, and HRV-derived autonomic indicators, this study provides empirical evidence on how feedback modality design shapes operator outcomes during human–cobot collaboration.

The findings reveal trade-offs between coordination efficiency, user experience, and physiological activation across different feedback modalities, highlighting feedback design as a key ergonomic parameter in collaborative robotic work systems.

This study makes three main contributions to research on human–cobot collaboration: (1) an experimental evaluation of feedback modalities within two collaboration configurations, (2) an integrated evaluation combining performance, subjective, and physiological measures, and (3) design-oriented guidelines for selecting feedback strategies in collaborative robotic work systems. To position this work within the broader research landscape, the following section reviews prior studies on human–robot collaboration, feedback modalities, and physiological assessment in interactive robotic systems.

## 2 Related work

### 2.1 Human–robot collaboration and interaction design

Human–robot collaboration (HRC) has increasingly been examined from an applied ergonomics perspective. In collaborative environments, the effectiveness of cobot deployment depends not only on mechanical capabilities but also on the design of interaction mechanisms that support human performance and wellbeing. Workstation-level design decisions such as task allocation, interaction timing, autonomy level, physical proximity, and communication mechanisms shape how humans and robots coordinate actions within shared workspaces (Villani et al., 2018; Bauer, 2016).

Empirical studies in industrially motivated assembly contexts demonstrate that collaboration configurations can significantly affect task completion time, error rates, workload, and operator acceptance (Fournier et al., 2024). Inadequate communication or unclear interaction cues may result in misunderstandings, reduced coordination efficiency, and increased cognitive demand (Taesi et al., 2023). Consequently, recent research emphasizes the importance of human-centered design principles when integrating collaborative robots into industrial environments, highlighting the need to align interaction mechanisms with operator cognitive capabilities and expectations (Ajoudani et al., 2018).

### 2.2 Feedback modalities and operator guidance in HRC

Effective human–robot collaboration often depends on clear communication of system state, task progress, and operator guidance. Feedback modalities such as auditory cues, visual displays, and multimodal combinations have been widely investigated as mechanisms for supporting coordination between human operators and robotic systems.

Auditory feedback can provide rapid attention-directing signals that require minimal visual attention, while visual displays allow more detailed representation of task-relevant information. Multimodal feedback combining auditory and visual cues may improve communication robustness and task comprehension, although it can also increase cognitive demand depending on task complexity and interaction context.

More recently, spatial guidance techniques, including projected assistive interface, have been explored as mechanisms for improving task transparency and coordination in collaborative workspaces (Sahoo and Biondi, 2026; Capponi et al., 2024). Such approaches highlight task-relevant spatial targets and may reduce visual search demands during collaborative tasks by directing the operator’s attention to relevant regions of the workspace.

However, empirical findings remain mixed, and the effectiveness of different feedback modalities appears to depend strongly on the coordination demands and interaction structure of the collaborative task. These observations highlight the need for systematic empirical comparisons of feedback modalities across different human–robot interaction contexts in order to inform interface design for collaborative robotic work systems.

### 2.3 Physiological measures in human–robot interaction

Beyond subjective questionnaires and task performance metrics, physiological measures have increasingly been employed to evaluate operator state in human–robot interaction and human–machine cooperation contexts (Charles and Nixon, 2019; Dehais et al., 2020). Physiological indicators can provide objective insights into autonomic regulation and may capture aspects of operator state that are not easily accessible through self-report measures. Several studies have demonstrated that physiological metrics are sensitive to cognitive demand, coordination complexity, and human–machine interaction workload, making them suitable indicators for assessing operator state in collaborative robotic tasks (Dehais et al., 2020; Charles and Nixon, 2019; Hopko et al., 2023).

Among these measures, HRV has emerged as a widely used indicator of autonomic nervous system activity. HRV-derived metrics have been used to assess workload, stress-related responses, and physiological arousal during interactive tasks. In human–robot collaboration research, physiological sensing has therefore been proposed as a promising tool for evaluating ergonomics and sustainable human performance in shared work environments (Gualtieri et al., 2021).

Recent studies indicate that interaction characteristics such as task demands, coordination complexity, and perceived system reliability can influence physiological responses during collaborative tasks (Hopko et al., 2023). Experimental studies in collaborative handover and coordination tasks further report measurable stress-related responses during human–robot cooperation (Su et al., 2024). However, many existing studies examine physiological indicators in isolation or within narrowly defined interaction scenarios. As a result, their findings are often difficult to translate into practical interface design decisions for collaborative robotic workstations where interaction conditions and feedback modalities may vary across tasks.

These limitations motivate further empirical investigation of how interaction design choices, such as feedback modality, influence both operator experience and physiological responses during human–robot collaboration.

Taken together, prior research highlights the importance of interaction design, feedback modality, and operator state in human–robot collaboration. However, empirical evidence remains limited regarding how different feedback modalities influence both subjective and physiological outcomes during collaborative tasks with varying coordination demands. This motivates the empirical investigation presented in the following sections.

## 3 Methodology

**FIGURE 1:**
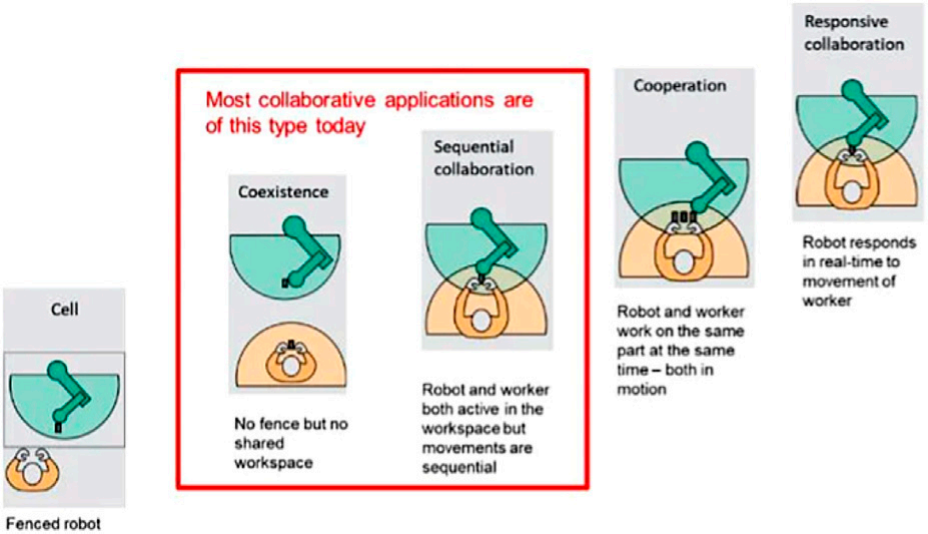
Conceptual levels of human-robot collaboration in industrial settings, synthesized based on prior literature (e.g. (Bauer, 2016; Villani et al., 2018)).

Figure 1 illustrates commonly referenced levels of human–robot collaboration in industrial settings, ranging from sequential turn-taking interactions to tightly coupled synchronous coordination. These conceptual levels reflect increasing demands on interaction transparency, feedback mechanisms, and mutual adaptation, and are often used to describe different coordination structures in collaborative work systems.

The present study draws on this conceptual framework to design two experimental interaction scenarios representing distinct coordination structures. In the first scenario, human and robot actions follow a sequential turn-taking pattern, whereas the second scenario requires continuous synchronous coordination between the operator and the cobot. Feedback modality effects are examined *within* each scenario rather than as a direct comparison between collaboration levels.

### 3.1 Experimental design

Two experimental scenarios representing different coordination structures were used. Each participant experienced the two experimental scenarios (described in detail in the following section). Scenario one included four feedback modalities (None, Auditory, Visual, Auditory + Visual), whereas Scenario two included five modalities (None, Auditory, Visual, Auditory + Visual, PAI).

The order of modalities was kept fixed within each scenario for all participants to ensure comparable exposure across conditions. This design choice was intended to maintain comparable task exposure and to reduce variability associated with uncontrolled transitions between modalities. By preserving a uniform sequence across participants, all individuals experienced identical contextual conditions, thereby supporting more direct comparisons between feedback modalities without additional confounding effects related to differing task histories.

Although counterbalancing and randomization are commonly used to reduce potential learning or fatigue effects, such approaches may also introduce additional between-subject variability, particularly in studies involving complex human–cobot interaction tasks. In the present study, participants required a certain degree of familiarization with the cobot and the collaborative task structure. A fixed progression therefore enabled participants to develop a more stable baseline of interaction competence before encountering subsequent modalities. This structured progression was intended to reduce the influence of initial unfamiliarity with the system on later conditions.

Furthermore, the primary objective of this study was to compare feedback modalities under consistent experiential conditions rather than to isolate modality effects entirely independent of interaction history. Using a fixed order allowed potential learning or fatigue effects to occur consistently across participants and modalities, rather than unevenly distributed as may occur under randomized sequencing. Although residual order-related effects cannot be fully excluded, the fixed sequence allowed any such effects to remain relatively consistent across participants and conditions. Nevertheless, short rest periods were implemented between conditions to reduce the potential effects of fatigue and adaptation. Future studies may further examine counterbalanced or randomized experimental designs to isolate order-related effects more explicitly.

Across both scenarios, we manipulated feedback modality as the primary independent variable, comparing the None, Auditory, Visual, and Auditory + Visual feedback modalities, and (in the high-collaboration configuration only) additional Projected Assistive Interface (PAI) feedback.

The PAI condition in this study was implemented as a screen-based assistive interface rather than a physically projected interface within the workspace. The term is retained for consistency with prior conceptual framing, but should be interpreted accordingly.

PAI feedback was implemented only in Scenario two because this task required continuous spatial coordination and real-time corrections, where spatially grounded guidance can reduce ambiguity and support shared control. In Scenario 1 (turn-taking), actions were temporally separated and guidance needs were discrete, so PAI was not expected to add task-relevant information beyond conventional visual or auditory cues. Restricting PAI to Scenario two also reduced experimental complexity and avoided conflating feedback effects with fundamentally different task structures. The two scenarios were designed to represent different coordination structures that may arise in human–robot collaborative work systems.

Based on the gaps identified in the literature, the present study addresses the following research questions:

- RQ1: How do different feedback modalities influence task performance in human–cobot collaborative tasks?
- RQ2: How do different feedback modalities influence operators’ subjective experience during human–cobot collaboration?
- RQ3: How do different feedback modalities influence operators’ autonomic physiological responses, as reflected in HRV-derived indicators, during human–cobot collaboration?

Table 1 summarizes the dependent and interdependent variables that were examined in the two scenarios.

**TABLE 1:** Summary of independent and dependent variables examined in the experiment.

Variable type	Variable	Description
Independent variable	Feedback modality	Four modalities in Scenario 1 (None, auditory, visual, Auditory + Visual) and five modalities in Scenario 2 (None, auditory, visual, Auditory + Visual, PAI)
Independent variable	Collaboration scenario	Two experimental interaction scenarios representing different coordination structures: (1) sequential turn-taking collaboration and (2) synchronous real-time coordination
Dependent variable	Task performance	Task completion time and task execution efficiency during the collaborative activity
Dependent variable	Subjective user experience	Self-reported measures including perceived workload (NASA-TLX), perceived safety, comfort, and trust in the cobot
Dependent variable	Physiological responses	Autonomic activity derived from HRV indicators including stress index, SNS and PNS indices, SDNN, RMSSD, and additional HRV metrics

### 3.2 Experimental procedure

#### 3.2.1 Scenario 1: low collaboration (turn-taking cooperation)

**FIGURE 2:**
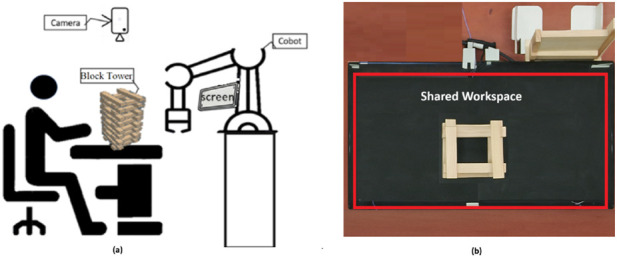
Low-collaboration (turn-taking) scenario. General cell setup **(a)** and top camera view of the shared workspace **(b)**.

In this scenario, the participant and the cobot alternately built a block tower using wooden blocks. The human and the cobot were positioned opposite each other at a shared worktable. The general cell layout and workspace configuration for this low-collaboration scenario are illustrated in Figure 2.

A vision-based hand tracking system, installed above, detected when the participant’s hand entered the shared workspace. Once the participant placed a block and withdrew her/his hand, the cobot was triggered to place the next block. This enforced turn-taking mechanism to ensure temporal separation of actions and supported consistent task execution. The participants were informed of their turn to place a new wooden block using one of the following feedback modalities:

- None: The participants inferred turn-taking from the cobot’s last action.
- Auditory: The cobot emitted an auditory cue (e.g., “Now it’s your turn”).
- Visual: The cobot displayed a visual message on the screen indicating the participant’s turn.
- Auditory + Visual: The cobot provided both auditory and visual cues simultaneously.

#### 3.2.2 Scenario 2: high collaboration (responsive, continuous synchronous collaboration)

**FIGURE 3:**
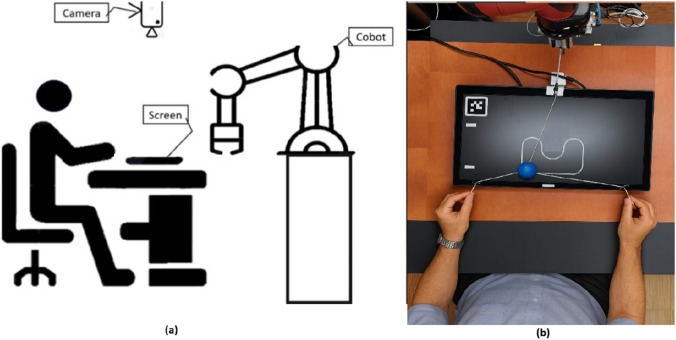
High-collaboration (synchronous) scenario. Ball alignment task using a string-tension interface: general cell setup **(a)** and top-camera view **(b)**.

In this scenario, the participant and cobot were required to jointly maneuver a ball along a projected path using a string-tension interface. The participant controlled the lateral (X-axis) movement, while the cobot controlled the longitudinal (Y-axis) movement in real time by pulling or releasing a string mechanically attached to its gripper. A camera mounted above the table monitored the ball’s position relative to a target path trajectory. If the ball deviated substantially from the target path, participants were required to correct its position before continuing along the trajectory, which could increase task completion time. The general cell layout and interaction setup for this high-collaboration scenario are illustrated in Figure 3.

This scenario represents a synchronous coordination task requiring continuous interaction between the human operator and the cobot, in which both agents act simultaneously toward a shared goal.

**FIGURE 4:**
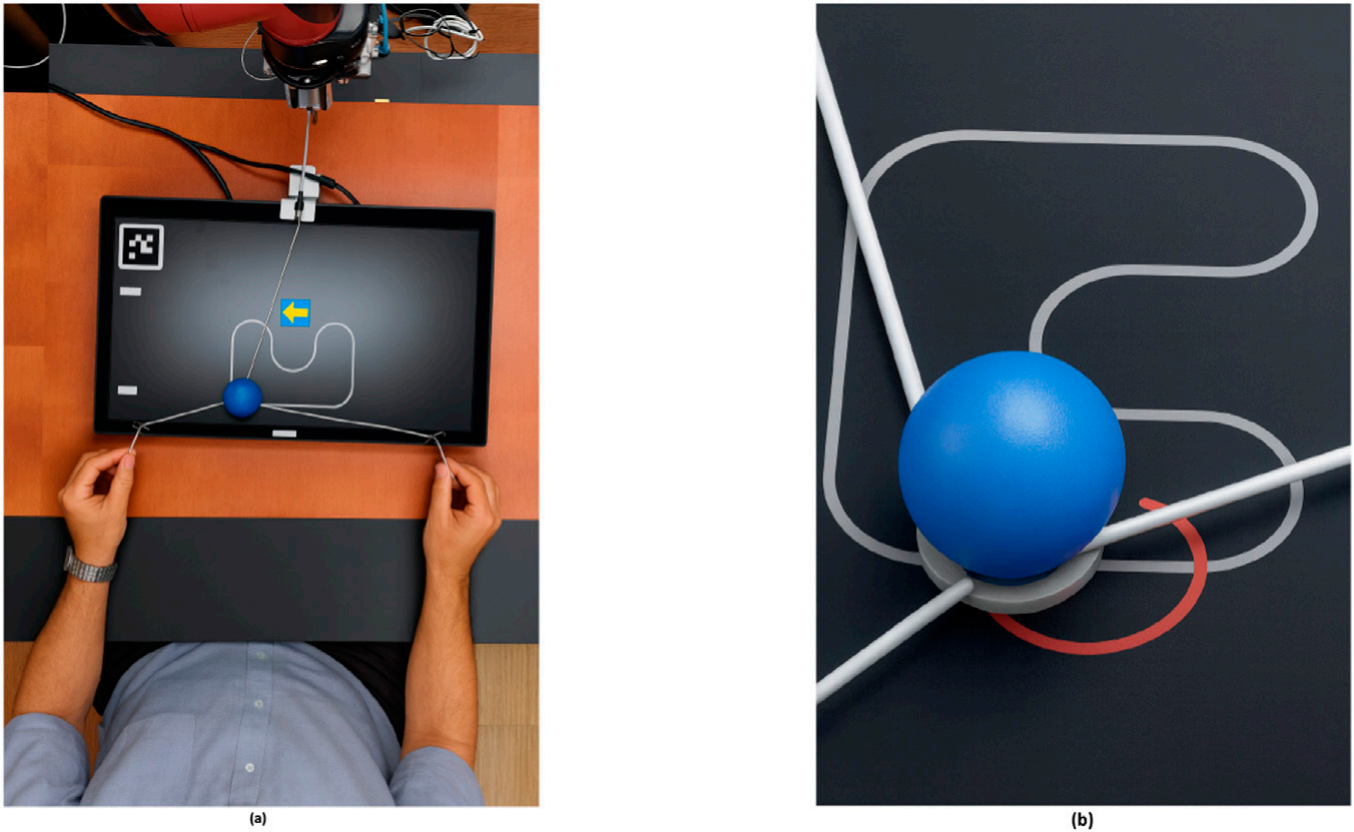
Feedback modalities in the high-collaboration scenario displayed on the tabletop screen. **(a)**Visual and PAI-based feedback modalities in the high-collaboration scenario, displayed on the tabletop screen. **(b)**PAI condition. A red circular marker highlights the next target region for ball positioning on the tabletop display.

The following feedback modalities were evaluated:

- None: Collaboration without additional cues beyond the inherent task dynamics.
- Auditory: The cobot provided auditory guidance (e.g., “Pull right”/“Pull left”) to support corrective actions.
- Visual: Screen-based visual cues were presented, including directional arrows indicating the required pulling direction and a circular icon signaling excessive string tension (Figure 4a).
- Auditory + Visual: Combined auditory and visual cues delivered simultaneously.
- PAI: A projected assistive interface cue highlighted the next desired target region for ball positioning using a red circular marker (Figure 4b).

The study included 79 participants (aged 22-28), all undergraduate students in Industrial Engineering and Management. Participants had no prior professional experience working with collaborative robots. This population was selected to represent novice operators with technical background but limited practical exposure to cobot-based workstations. Written informed consent was obtained from all participants prior to participation in the study.

The experimental tasks were designed to be intuitive and did not require prior training in robot programming or operation. While the use of a student population may limit direct generalization to experienced industrial workers, it allows for controlled comparison of collaboration configurations and interaction modalities under consistent baseline conditions.

Each participant completed four repetitions per feedback condition within each scenario. A 2-min resting period was implemented between conditions to support physiological recovery and reduce carry-over effects. For statistical analysis, performance, subjective, and physiological measures obtained across the four repetitions within each feedback condition were aggregated by computing a mean value per participant and condition prior to inferential testing. This aggregation approach was adopted to reduce trial-level variability and to enable within-subject comparisons across feedback modalities at the participant level.

**FIGURE 5:**
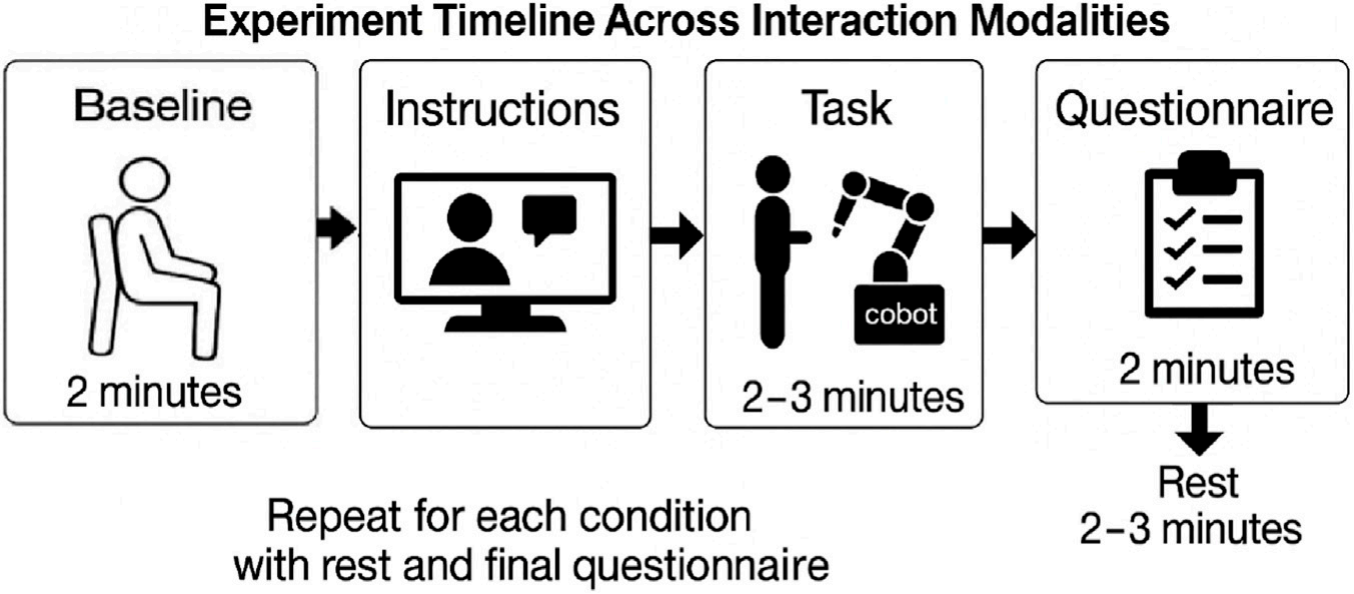
Experimental timeline within each collaboration scenario, illustrating baseline recording, task execution across feedback modalities, rest periods between conditions, and post-scenario questionnaire administration.

Figure 5 summarizes the experimental timeline within each collaboration scenario. Each trial began with a baseline recording phase to capture resting physiological activity, followed by short written and oral instructions. Next, participants performed the collaborative task under the corresponding feedback modality. After each trial, a structured rest period was introduced before proceeding to the next modality in order to support physiological recovery and reduce carry-over effects. After completing all conditions within a scenario, participants completed a post-scenario questionnaire that included separate rating sections for each feedback modality experienced during that scenario. Participants retrospectively evaluated each modality individually with respect to task understanding, comfort, interaction quality, trust, and related subjective measures. This standardized sequencing was applied consistently across both scenarios to enable comparison of subjective and physiological measures across feedback conditions.

### 3.3 Apparatus and measures

The collaborative robot used in the study was a Sawyer cobot (Rethink Robotics), a 7-degree-of-freedom robotic manipulator for safe human-robot collaboration. Visual, auditory, and projected assistive interface (PAI) feedback were delivered using a workstation-mounted display and speakers. The feedback system was placed horizontally on the worktable, allowing participants to receive visual and PAI-based guidance while maintaining visual access to the physical workspace and the collaborative task. In the PAI condition, guidance cues were presented on the screen as spatially referenced visual overlays aligned with the task progression. The examined PAI configuration was designed specifically for the high-collaboration task in Scenario two and represents a task-tailored interface approach based on computer-generated guidance signals adapted to the coordination demands of complex collaborative interaction. A vision system mounted above the workspace was used to track task-relevant objects.

We combined objective physiological indicators with subjective self-report measures to characterize operator state during human-cobot collaboration. Subjective experience was assessed using standardized self-report instruments. Perceived workload was measured using the NASA Task Load Index (NASA-TLX), and user’s attitudes toward robots were assessed using the “Negative Attitudes Toward Robots Scale” (NARS) (Nomura et al., 2006). In addition, perceived stress and trust were measured using direct post-task questionnaire items (Supplementary Appendix A). Objective physiological responses were operationalized using standard HRV indices, including SDNN, RMSSD, stress index, SNS and PNS indices, mean heart rate, and pNNxx measures (see Table 2 for details). These indices were selected because they provide a validated, non-invasive, and widely used set of markers for autonomic regulation and stress-related responses during interactive tasks (Shaffer and Ginsberg, 2017; Brusaca et al., 2021).

**TABLE 2:** Physiological metrics used in the study and their typical association with autonomic regulation.

Metric	Interpretation
SDNN	Global HRV index reflecting overall autonomic flexibility; lower values are commonly associated with increased strain
RMSSD	Short-term HRV marker of parasympathetic (vagal) activity; lower values indicate reduced parasympathetic regulation
Stress index	Composite Kubios-derived indicator of sympathetic dominance; higher values reflect increased physiological stress
SNS index	Index of sympathetic nervous system activation; higher values indicate increased arousal
PNS index	Index of parasympathetic activity; more negative values indicate reduced parasympathetic contribution
Mean HR	Average heart rate during task execution; elevated values may reflect increased arousal or workload
Max and min HR	Range of heart rate extremes; larger excursions indicate stronger autonomic reactivity
pNNxx	Percentage of adjacent NN intervals differing by more than 50 m (pNN50); higher values reflect greater beat-to-beat variability and parasympathetic activity

Internal consistency of composite scales was evaluated using Cronbach’s α . Likert-type composite scores were treated as approximating interval-level data and analyzed using parametric statistical tests.

A one-way repeated-measures ANOVA was conducted separately for each scenario to evaluate the effect of feedback modality, followed by Bonferroni-adjusted pairwise comparisons.

This approach is commonly adopted when aggregated scales demonstrate acceptable internal consistency, though it relies on assumptions that may not fully hold for ordinal responses. In line with best-practice recommendations (Schrum et al., 2023), reliability metrics are reported, and the limitations of this approach are acknowledged.

HRV was prioritized as the primary physiological channel because it provides a reliable and minimally intrusive index of autonomic regulation during task performance and is relatively robust to moderate body movement compared with electrodermal activity measures (Shaffer and Ginsberg, 2017; Laborde et al., 2017). Although electrodermal activity (e.g., GSR) is frequently used for stress assessment, it is particularly sensitive to motion artifacts and contact-pressure effects. Given that both experimental scenarios required continuous hand and arm movements, HRV was deemed more robust than GSR for the present tasks.

Physiological data were collected using a Polar H10 heart rate sensor, which provides high-resolution inter-beat interval (IBI) measurements suitable for HRV analysis. The sensor was worn by participants throughout the experiment and synchronized with task execution to capture autonomic responses during each interaction condition.

Physiological data were processed using Kubios Premium software (Tarvainen et al., 2014). A standard short-term HRV analysis configuration was applied consistently across participants and experimental conditions, including detrending using the smoothness priors method (lambda = 500), artifact correction with an automatic medium threshold, and interpolation at 4 Hz.

In addition, all recordings were visually inspected to ensure signal quality and to verify the effectiveness of artifact correction.

Frequency-domain metrics were computed using default band definitions (LF: 0.04–0.15 Hz; HF: 0.15–0.40 Hz). Full HRV processing parameters are provided in Supplementary Appendix B. HRV analysis was performed on task segments corresponding to each experimental condition, with segment durations ranging approximately between 80 and 130 s, depending on task completion time. While these durations are shorter than standard long-term HRV recordings, short-term HRV analysis is commonly used to capture relative changes in autonomic activation across experimental conditions rather than absolute physiological states. Given the relatively short duration of task segments, frequency-domain HRV metrics should be interpreted with caution, as shorter windows may limit the stability of low-frequency components. Accordingly, these metrics are considered supplementary indicators of autonomic trends rather than definitive measures. HRV metrics were computed per condition using the corresponding task segment and were normalized by comparing each condition to a pre-task resting baseline for each participant.

To assist interpretation of the physiological metrics reported throughout the manuscript, Table 2 summarizes the primary HRV and cardiovascular indicators used in this study and their typical association with stress and autonomic regulation.

## 4 Results

This section presents the experimental results obtained in the two collaboration scenarios. Results are reported separately for each scenario, with statistical comparisons conducted across feedback modalities within the same collaboration configuration. We first report subjective outcomes derived from the post-scenario questionnaires, followed by objective task performance measures and HRV-based physiological responses. Statistical comparisons across feedback modalities are then summarized to support interpretation of the findings. All statistical comparisons were conducted within each scenario.

### 4.1 Summary of self-reported measures

The questionnaires captured general background information, as well as subjective assessments of task understanding, comfort, and interaction quality after each collaboration scenario. Questionnaire-based subjective measures were analyzed separately for each feedback modality within the corresponding collaboration scenario. For clarity, the inferential labels reported later in the statistical summaries correspond directly to specific questionnaire-based subjective measures and participants’ self-perceived interaction experience. In particular, “user experience rating” refers primarily to participants’ self-evaluation of comfort, perceived task understanding, and overall interaction experience, whereas “interaction quality rating” refers to perceived collaboration quality, trust, and coordination-related interaction assessments associated with each feedback modality.

Standardized multi-item questionnaires demonstrated acceptable internal consistency. Cronbach’s α values were 0.81 for the NASA-TLX scale and 0.79 for the NARS scale. Positive response percentages for modality-specific subjective measures were calculated as the proportion of participants selecting positive agreement ratings for each questionnaire item and feedback modality condition.

#### 4.1.1 Participant background questionnaire

Familiarity with collaborative robots. A total of 55.7% of participants reported that they were unfamiliar with the term “collaborative robot”. However, 81% indicated prior exposure to robotic systems or automation technologies, primarily through academic coursework. This indicates that while formal terminology was not widely recognized, most participants reported prior experience with robots in educational contexts.

Attitudes toward collaboration with cobots. More than 60% of participants reported that they did not feel fear or hesitation when collaborating with the cobot. Responses were distributed across the scale, with most participants reporting neutral to positive ratings.

Concerns regarding the broader impact of cobots. Participants rated five statements addressing perceived social and emotional implications of increased cobot deployment, yielding total scores ranging from 5 to 25 where five represents no concerns and 25 very high concerns. Approximately 86% of the participants reported scores between 11 and 22, indicating moderate to high levels of concern regarding the broader implications of collaborative robot adoption.

Concerns during human-cobot collaboration. Participants responded to three items related to emotional responses during interaction, using a one to five rating scale (total range: 3-15). Approximately 75% of participants reported scores between 5 and 10, indicating low to moderate concern levels during direct collaboration with the cobot.

#### 4.1.2 Self-reported interaction quality and comfort in scenario one

Table 3 presents participants’ self-assessed understanding of the task and their perceived comfort during the low-collaboration (turn-taking) task. As shown, the combined auditory + visual feedback received the highest score of both understanding and comfort.

**TABLE 3:** Self-reported task understanding and comfort across feedback modalities in Scenario 1 (low collaboration). Percentages represent the proportion of participants reporting positive ratings (above the midpoint of the Likert scale) for each measure.

Feedback modality	% Understanding	% Comfort
Non-feedback	65.8%	53.2%
Visual feedback	56.9%	45.6%
Auditory feedback	68.4%	59.5%
Auditory + visual feedback	77.2%	64.6%

#### 4.1.3 Self-reported interaction quality and comfort in scenario two

Participants’ self-assessed understanding of the task and their perceived comfort during the high-collaboration (synchronous) scenario are summarized across the five feedback modalities, including projected assistive interface (PAI), and shown in Table 4.

**TABLE 4:** Self-reported task understanding and comfort across feedback modalities in Scenario 2 (high collaboration). Percentages represent the proportion of participants reporting positive ratings (above the midpoint of the Likert scale) for each measure.

Feedback modalities	% Understanding	% Comfort
Non-feedback	40.5%	32.9%
Visual feedback alone	42.7%	42.7%
Auditory feedback alone	46.8%	53.2%
Visual and auditory feedback	51.9%	58.2%
Projected assistive interface (PAI) feedback	61.6%	77.2%

Reported understanding varied across conditions, with the highest score observed under the PAI feedback modality (61.6%). Comfort ratings showed a similar pattern, with PAI yielding the highest reported comfort (77.2%).The lowest scores of reported understanding and comfort were observed in the no-feedback condition.

### 4.2 Task completion time

Mean completion times across all feedback modalities were measured for each condition and are presented separately for the low- and high-collaboration tasks.

#### 4.2.1 Scenario 1 – low collaboration task

**FIGURE 6:**
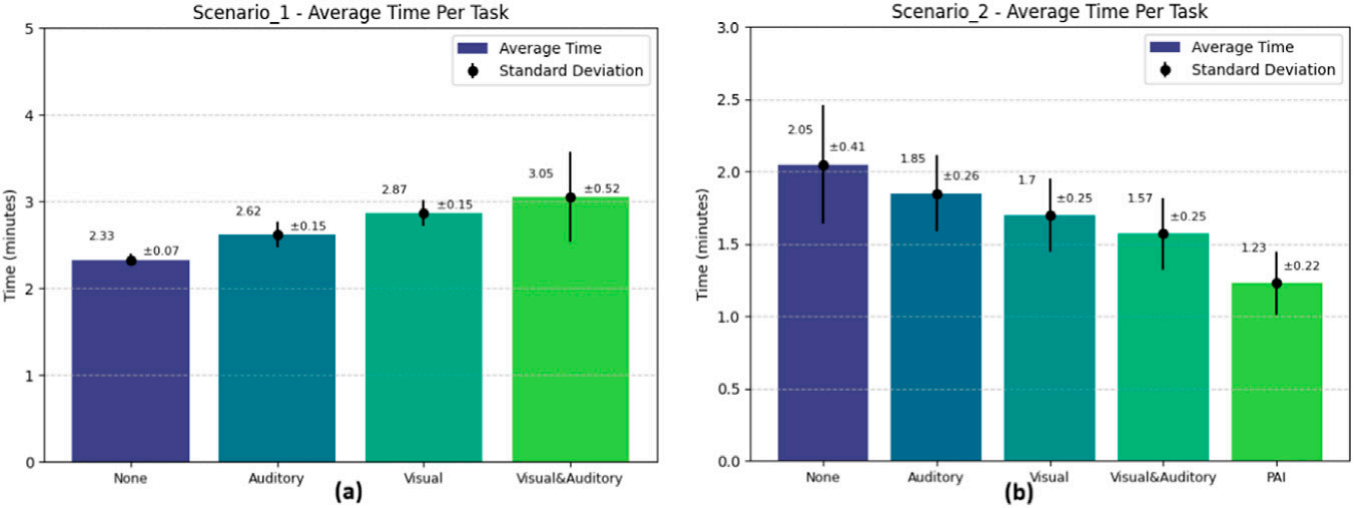
Mean task completion times for **(a)** low-collaboration and **(b)** high-collaboration tasks across feedback modalities.

As shown in Figure 6a, the no-feedback condition exhibited the shortest mean completion time (2.33 min), while the combined auditory and visual feedback condition exhibited the longest mean completion time (3.05 min).

#### 4.2.2 Scenario 2 – high collaboration task

For the high-collaboration scenario (Figure 6b, the PAI feedback condition exhibited the shortest mean completion time (1.23 min), whereas the no-feedback condition exhibited the longest mean completion time (2.05 min).

### 4.3 Physiological results in scenario 1 (low collaboration)

**FIGURE 7:**
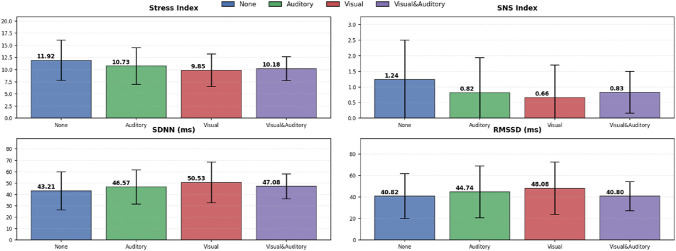
Primary HRV indicators across feedback modalities in Scenario 1 (low-collaboration turn-taking task). Values represent mean ± SD across participants.

To address RQ3, autonomic activity during the collaborative task was assessed using HRV indices derived from Kubios analysis. Figure 7 presents the primary HRV indicators obtained across the four feedback modalities in Scenario 1.

Overall, the observed differences in autonomic activity across feedback conditions were relatively modest. The no-feedback condition exhibited the highest mean stress index value (11.92), whereas lower values were observed in the visual (9.85) and combined auditory–visual (10.18) conditions. Similarly, sympathetic nervous system (SNS) index values were highest in the no-feedback condition (1.24) and lowest in the visual feedback condition (0.66).

Time-domain HRV measures also varied across feedback modalities. SDNN values were highest in the visual feedback condition (50.53 ms) and lowest in the no-feedback condition (43.21 ms). RMSSD values were likewise highest in the visual feedback condition (48.08 ms) and lowest in the combined auditory–visual condition (40.80 ms).

These results indicate modest variability in autonomic activity across feedback modalities in the turn-taking collaboration scenario. Descriptive statistics for the primary HRV indicators are illustrated in Figure 7. Complete descriptive statistics for all physiological measures, including additional HRV metrics not shown in the main figures, are provided in Supplementary Appendix C.

To assess statistical differences across feedback modalities in Scenario 1, repeated-measures ANOVA followed by Bonferroni-adjusted *post hoc* comparisons were conducted for all physiological measures. Detailed statistical results are reported in Section 4.5.

### 4.4 Physiological results in scenario 2 (high collaboration)

**FIGURE 8:**
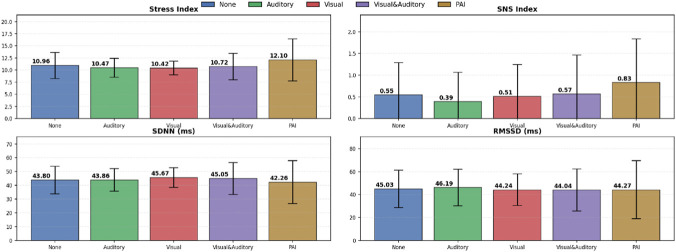
Primary HRV indicators across feedback modalities in Scenario 2 (high-collaboration synchronous coordination task). Values represent mean ± SD across participants.

Figure 8 presents the primary HRV indicators obtained across the five feedback modalities in the synchronous collaboration scenario.

Across the conditions, the projected assistive interface (PAI) feedback modality exhibited the highest mean stress index value (12.10), whereas the visual feedback condition showed the lowest value (10.42). Intermediate stress index values were observed for the auditory–visual (10.72) and auditory-only (10.47) conditions.

Similarly, sympathetic nervous system (SNS) index values were highest in the PAI condition (0.83) and lowest in the auditory-only condition (0.39). The remaining feedback modalities showed intermediate SNS values.

Time-domain HRV measures showed relatively modest variability across feedback modalities. SDNN values were highest in the visual feedback condition (45.67 ms) and lowest in the PAI condition (42.26 ms). RMSSD values were highest in the auditory-only condition (46.19 ms) and lowest in the combined auditory–visual condition (44.04 ms).

Descriptive statistics for the primary HRV indicators are illustrated in Figure 8. Complete descriptive and inferential statistics for all physiological measures, including all HRV-derived metrics (both statistically significant and non-significant), are provided in Supplementary Appendix C to ensure full transparency of the analysis.

### 4.5 Statistical analysis summary

Statistical analyses were conducted to evaluate differences across feedback modalities for both collaboration scenarios.

Given the within-subject design, in which each participant experienced multiple feedback modalities, repeated-measures ANOVA was applied with interaction modality as a within-subject factor. Assumptions of sphericity were evaluated using Mauchly’s test, and where violations were detected, Greenhouse–Geisser corrections were applied. Effect sizes (partial $\eta^{2}$ ) are reported for all main effects.

Subjective questionnaire outcomes included both standardized multi-item scales and modality-specific single-item ratings, analyzed separately within each collaboration scenario. NASA-TLX and NARS scores were computed according to their standard scoring procedures. In addition, several modality-specific subjective outcomes, including perceived task understanding, comfort, trust, safety, and collaboration quality, were evaluated using individual questionnaire items corresponding directly to each construct. These ratings were analyzed separately for each feedback modality within the corresponding scenario. Only questionnaire items directly relevant to the reported experimental comparisons were included in the statistical analyses presented in the Results section.

#### 4.5.1 Scenario 1 – low collaboration task

For Scenario 1, interaction modality (None, Auditory, Visual, Auditory + Visual) was analyzed as the within-subject factor. Dependent variables included task completion time, questionnaire-derived subjective measures related to user experience and interaction quality, and physiological HRV measures. The subjective measures reflected participant ratings of comfort, perceived task understanding, trust, collaboration quality, and coordination-related interaction experience across feedback modalities. However, no statistically significant differences between feedback modalities were observed across the physiological indicators (p>.05) .

A repeated-measures ANOVA with Greenhouse–Geisser correction revealed a significant effect of interaction modality on task completion time, F(2.35,192.82)=692.53 , p<.001 , $\eta_{p}^{2}=0.894$ . Mauchly’s test indicated that the assumption of sphericity was violated (p<.001) , and therefore corrected results are reported. Pairwise comparisons with Bonferroni adjustment indicated multiple statistically significant differences between modality pairs. Task completion time showed a progressive increase across conditions, from the no-feedback condition (M = 2.33 min), to auditory (M = 2.62 min), to visual (M = 2.87 min), and reached its highest values in the combined visual–auditory condition (M = 3.05 min).

A summary of statistically significant comparisons is reported in Table 5.

**TABLE 5:** Pairwise comparisons with Bonferroni adjustment for Scenario 1 (significant results only).

Dependent variable	Significant comparison	*p*-value
Task completion time	None vs. Auditory + Visual	<0.001
	None vs. Visual	0.002
	None vs. Auditory	0.007
User experience rating	Visual vs. Auditory	0.015
	Visual vs. Auditory + Visual	0.028
	Visual vs. None	0.030
Interaction quality	Visual vs. Auditory + Visual	0.021
	Visual vs. None	0.042

#### 4.5.2 Scenario 2 – high collaboration task

For Scenario 2, repeated-measures ANOVA was performed with interaction modality (None, Auditory, Visual, Auditory + Visual, PAI) as a within-subject factor. Dependent variables included task completion time, self-reported interaction quality, and physiological measures derived from HRV analysis.

A repeated-measures ANOVA with Greenhouse–Geisser correction revealed a significant effect of interaction modality on task completion time, F(2.54,203.51)=56.83 , p<.001 , $\eta_{p}^{2}=0.415$ . Mauchly’s test indicated that the assumption of sphericity was violated (p<.001) , and therefore corrected results are reported. Pairwise comparisons with Bonferroni adjustment indicated multiple statistically significant differences between modalities. Task completion time decreased progressively across conditions, from the no-feedback condition (M = 2.05 min), to auditory (M = 1.85 min), to visual (M = 1.7 min), to combined visual–auditory feedback (M = 1.57 min), and was lowest in the PAI condition (M = 1.23 min). Notably, the PAI condition yielded a substantial and consistent performance improvement across participants compared to all other modalities.

A summary of statistically significant comparisons is presented in Table 6.

**TABLE 6:** Bonferroni-adjusted pairwise comparisons for Scenario 2 (significant results only).

Dependent variable	Significant comparison	*p*-value
Task completion time	PAI vs. None	<0.001
	PAI vs. Auditory	<0.001
	PAI vs. Visual	<0.001
	PAI vs. Auditory + Visual	<0.001
	None vs. Auditory	<0.001
	None vs. Visual	<0.001
	None vs. Auditory + Visual	<0.001
User experience rating	PAI vs. None	<0.001
	PAI vs. Auditory	<0.001
	PAI vs. Visual	<0.001
	PAI vs. Auditory + Visual	<0.001
	None vs. Auditory	<0.001
	None vs. Visual	<0.001
	None vs. Auditory + Visual	<0.001
Interaction quality	PAI vs. None	0.034
	PAI vs. Visual	0.034
	None vs. Auditory	0.045
	None vs. Auditory + Visual	0.045
Stress index	PAI vs. Visual	0.027
	None vs. Visual	0.039
SNS index	PAI vs. Auditory	0.044
PNS index	Auditory vs. PAI	0.032
pNNxx	Auditory vs. Auditory + Visual	0.041

## 5 Discussion

The discussion focuses on how feedback modality shapes coordination, perceived safety, and operator state in the two human–cobot tasks with different collaboration structures. The results from the two collaboration scenarios indicate that the effectiveness of feedback modalities depends strongly on the coordination structure of the task, influencing task performance, subjective experience, and physiological activation in different ways. In particular, the results obtained with the projected assistive interface (PAI) highlight the importance of spatially integrated feedback for supporting efficient human–robot coordination. Although these findings are limited to the specific characteristics of Scenario 2, they may suggest the potential value of task-tailored interface approaches in complex high-collaboration human–robot interaction settings. We emphasize design trade-offs between efficiency, user experience, and physiological activation, and translate the findings into design-oriented guidance for collaborative work systems.

### 5.1 Collaboration configuration as a design parameter

The findings highlight the importance of treating human-robot collaboration configuration as an explicit and adjustable design parameter in industrial workstations, rather than as a fixed outcome of safety constraints or task allocation decisions. Across the two tasks with distinct coordination structures, we observed that patterns of performance, subjective experience, and physiological activation varied systematically with task demands.

In the low-collaboration scenario, interaction was temporally separated and highly structured, with no statistically significant modulation of HRV-derived indices by feedback modality. However, reduced collaboration did not consistently correspond to reduced operator strain. In Scenario 1, the no-feedback condition showed descriptively higher stress-related indices and peak heart rate, despite its simpler interaction structure and the absence of additional feedback modalities.

By contrast, the high-collaboration scenario required continuous, real-time coordination between the human operator and the cobot within a shared workspace. This configuration showed descriptively higher autonomic activation across several HRV-derived indices, alongside improved task performance under feedback modalities that supported timely guidance and coordination. These results suggest that elevated physiological activation in high-collaboration contexts does not necessarily reflect detrimental stress, but may instead be consistent with engagement-related arousal under the specific coordination demands of the present task, particularly when accompanied by improved task performance and positive subjective experience.

These findings suggest that collaboration intensity should not be minimized by default to reduce interaction frequency or physical proximity. Instead, collaboration configuration should be selected in relation to task requirements, operator capabilities, and the availability of effective feedback mechanisms. High-collaboration configurations may be advantageous for tasks that benefit from shared control and continuous coordination, provided that transparency and feedback timing are carefully designed. Conversely, low-collaboration configurations may be appropriate for simpler or highly standardized tasks, but should not be assumed to reduce operator strain in the absence of sufficient interaction cues.

Overall, the results suggest that collaboration configuration may function as a tunable system parameter; however, these findings should be interpreted within the specific experimental context, as differences between scenarios may also reflect variations in task structure, interaction demands, and potential sequence-related adaptation effects beyond collaboration intensity alone.

### 5.2 Differentiating physiological arousal from detrimental stress

The physiological results emphasize the importance of distinguishing context-dependent autonomic activation from responses that may reflect detrimental stress. Across both scenarios, HRV-derived indices showed variation across feedback conditions. These variations should be interpreted in light of the statistical results, which indicated that HRV differences across modalities were not statistically significant in Scenario one and only partially significant in Scenario 2. However, these physiological patterns did not consistently co-occur with negative subjective experience or degraded task performance, reinforcing the need for context-dependent interpretation.

In Scenario 1, the no-feedback condition showed descriptively higher stress-related indices and peak heart rate. Because HRV-based differences across modalities were not statistically significant, this pattern is interpreted cautiously. Still, the trend is consistent with the idea that reduced transparency can increase uncertainty and cognitive effort (inferring task state and timing), which may manifest as autonomic activation without corresponding benefits in performance or interaction quality. The absence of statistically significant HRV differences in the turn-taking scenario may be explained by the relatively low coordination demands of this task. Because human and robot actions were temporally separated, participants were not required to continuously monitor or adapt to the robot’s actions. As a result, the task likely imposed limited cognitive and coordination load, leading to relatively stable autonomic responses across feedback modalities.

In Scenario 2, on the other hand, several HRV-derived indices indicated higher autonomic activation under feedback conditions that also supported improved task performance and higher self-reported comfort, particularly in the projected assistive interface (PAI) and combined feedback modalities. Although these conditions elicited higher stress-related and sympathetic activation patterns (e.g., stress index and SNS index), they were simultaneously associated with faster task completion and more positive subjective evaluations. This convergence suggests that, within the specific coordination demands of the present scenario, autonomic activation may reflect task-related engagement in addition to stress-related processes, rather than serving as a direct or uniform indicator of detrimental stress. Interestingly, a comparable level of stress-related activation was also observed in the no-feedback condition of the turn-taking (Scenario 1). While PAI in the synchronous task likely increased physiological activation due to intensified coordination demands and information flow, the elevated stress index in the no-feedback condition may reflect uncertainty regarding task timing and role transitions. This observation suggests that autonomic activation in HRC may arise both from information overload and from insufficient interaction transparency, although these patterns were observed in different task contexts.

These findings align conceptually with perspectives in human-centered automation and neuro-ergonomics, which emphasize that physiological activation during human-machine interaction is best interpreted relative to task demands and concurrent subjective experience (Parasuram et al., 2006; Dehais et al., 2020). Interpreting all elevations in autonomic activity as negative stress risks oversimplifies the relationship between physiology and performance; moderate increases may instead reflect adaptive mobilization of cognitive and motoric resources during demanding collaborative tasks.

From a design and evaluation standpoint, treating any autonomic activation as undesirable may lead to suboptimal design decisions, for example, by discouraging forms of guidance that support effective coordination in demanding tasks. Conversely, identifying physiological activation patterns that coincide with uncertainty, limited feedback, or degraded performance can help detect interaction configurations that impose unnecessary cognitive or emotional burden. The combined use of physiological and subjective measures therefore enables a more nuanced assessment of whether observed arousal reflects functional engagement or emerging strain in collaborative manufacturing environments. These observations reinforce the importance of interpreting physiological signals within the broader interaction context, rather than treating autonomic activation as a direct proxy for negative stress in collaborative work systems.

### 5.3 The role of feedback modality in managing cognitive load and coordination

The comparative analysis of feedback modalities across both collaboration scenarios highlights feedback design as an important factor in shaping coordination quality, perceived safety, and operator experience during human-cobot collaboration. While feedback is often introduced to improve safety or task efficiency, the present results indicate that its effectiveness depends on how well the modality aligns with the temporal structure and coordination requirements of the task.

In the low-collaboration scenario, feedback primarily served a signaling and confirmation function, supporting turn-taking clarity and reducing uncertainty. Auditory and combined auditory-visual feedback were associated with higher self-reported comfort and interaction quality compared to visual-only feedback. This pattern suggests that for discrete, temporally separated actions, modalities that do not require sustained visual attention may support interaction without adding unnecessary monitoring demands. Visual-only feedback, in contrast, appeared less effective in this context, potentially because it required additional visual attention without clear informational gain.

In the high-collaboration scenario, feedback modality played a more critical role in supporting real-time coordination between the human and the cobot. Projected assistive interface (PAI) feedback enabled participants to align their actions with the cobot more efficiently, resulting in the shortest task completion times and the highest levels of perceived comfort and task understanding. These performance benefits indicate that spatially grounded, visually salient guidance can reduce ambiguity and support precise coordination in shared-control tasks. The results obtained with the projected assistive interface (PAI) suggest that it may serve as a coordination-support approach within the specific experimental configuration and interaction conditions examined in the present study, and may provide design-oriented insights for the development of other task-tailored interfaces in high-collaboration human–robot interaction settings. By integrating task-relevant guidance directly within the user’s visual workspace, the implemented PAI-based guidance appeared to support more intuitive and context-aware interaction under the coordination demands of the present task. Although the present findings are limited to the examined experimental configuration, future research may investigate whether similarly tailored interfaces can support other complex tasks that require high levels of human–cobot collaboration, including scenarios involving dynamic task allocation, adaptive assistance, and shared autonomy. Future research should therefore investigate the generalizability of PAI-based interaction across different task domains, as well as its long-term effects on user performance, trust calibration, and perceived safety in sustained human–robot collaboration. It should be noted that the PAI guidance implemented in this study was screen-based rather than head-mounted or spatially projected PAI, which may influence the level of visual immersion and associated cognitive demand.

However, the performance improvements associated with PAI were accompanied by elevated physiological activation, including higher stress index values and increased sympathetic activation. This pattern is consistent with the possibility that highly salient visual guidance supports coordination while also increasing cognitive and perceptual demands. However, the PAI implementation in the present study relied on screen-based overlays rather than fully spatially registered projected assistive interface (PAI), and therefore the observed effects should be interpreted within this interface configuration. In contrast, auditory-only feedback in Scenario two showed a comparatively favorable profile, with higher heart-rate stability (pNNxx) relative to the combined condition and lower SNS activation relative to PAI, suggesting that auditory cues can support coordination while preserving visual resources for monitoring the physical workspace.

Taken together, these findings suggest a trade-off in high-collaboration tasks: spatially integrated guidance can enhance coordination efficiency, but may be accompanied by increased physiological activation. More broadly, no single feedback modality is universally optimal across collaboration contexts. Feedback effectiveness depends on the interaction between task structure, collaboration intensity, and modality-specific demands. Visually intensive feedback such as PAI may be well suited for short-duration, precision-critical tasks where performance gains justify increased demands, whereas auditory or multimodal feedback may be preferable for sustained collaboration where minimizing cumulative strain and preserving operator comfort are critical.

These findings underscore the importance of treating feedback modality as an integral component of collaboration design rather than a secondary interface feature. Aligning feedback modality with task demands and collaboration configuration is essential for achieving effective coordination while maintaining manageable operator demands in industrial human-robot collaboration.

### 5.4 Implications for sustainable human performance in collaboration

The findings contribute to an applied ergonomics understanding of sustainable human performance in collaborative work systems, where performance must be balanced against longer-term operator wellbeing and system robustness. The observed patterns across collaboration configurations and feedback modalities indicate that sustainable performance cannot be inferred solely from short-term efficiency or immediate performance gains.

In particular, the results suggest that elevated physiological activation does not inherently indicate detrimental outcomes. In Scenario 2, some feedback conditions elicited higher autonomic activation while also supporting improved task performance and more positive subjective experience. This pattern is consistent with the possibility that, under appropriate support and transparency, autonomic activation may be consistent with task engagement under the specific experimental conditions rather than directly indicating harmful stress. From a sustainability perspective, this pattern may be consistent with task-related engagement that coincided with maintained coordination quality and positive subjective evaluations under the experimental conditions examined in the present study.

At the same time, the results point to configurations in which autonomic activation may reflect emerging strain rather than productive engagement. In Scenario 1, the no-feedback condition showed descriptively higher stress-related indices, but HRV-based differences across modalities were not statistically significant. Still, this directional pattern highlights a practical risk: even in temporally structured, low-intensity collaboration, reduced interaction transparency can increase uncertainty and cognitive effort, which may accumulate over repeated cycles and contribute to fatigue, reduced trust calibration, or lower acceptance over time.

From an industrial work-systems perspective, incorporating sustainability considerations into collaboration design has implications for training, shift-level performance stability, and workforce retention. Collaborative robotic systems that align task complexity with appropriate levels of feedback and interaction transparency are more likely to support consistent performance across time and reduce the risk of cumulative strain. Overall, the results support treating human performance as a dynamic system property that evolves in response to collaboration design, and reinforce the value of combining physiological and subjective indicators when evaluating sustainable collaboration in industrial settings.

### 5.5 Design-oriented guidelines for industrial deployment

Building on the empirical findings and their interpretation, we synthesize design-oriented guidelines for deploying collaborative robots in industrial work systems. These guidelines are not prescriptive rules, but evidence-informed principles intended to support practical decisions during system design, integration, and commissioning.

#### 5.5.1 Treat collaboration configuration as a potentially tunable design variable

The findings suggest that both low- and high-collaboration configurations can be viable when interaction transparency and feedback are aligned with task coordination demands. Rather than defaulting to minimal interaction to reduce perceived risk, designers should support adjustable collaboration modes (e.g., turn-taking vs. time-coupled shared control) that can be matched to task complexity and operator capability.

#### 5.5.2 Match feedback modality to the temporal structure of the task

In discrete, turn-taking tasks, guidance should primarily reduce uncertainty about role switching and timing, preferably using cues that do not impose sustained visual attention. In time-coupled coordination tasks, richer guidance (including spatially grounded cues) can improve alignment and efficiency, but should be applied selectively because it may increase cognitive demand and physiological activation.

#### 5.5.3 Prioritize transparency through anticipatory cues and clear state communication

Across collaboration modes, predictable robot behavior, explicit intent signaling, and unambiguous role allocation are central for perceived safety and calibrated trust. This is especially critical in shared workspaces and time-coupled collaboration, where uncertainty about timing or intent can impose avoidable cognitive effort.

#### 5.5.4 Evaluate guidance using multi-method evidence, not performance alone

Because performance gains can co-occur with elevated autonomic activation (as observed under some high-guidance conditions), evaluation should combine objective task metrics with subjective experience measures and, when feasible, physiological indicators. This supports identifying configurations that improve efficiency at the cost of increased strain.

#### 5.5.5 Use physiological measures as a commissioning and refinement tool

HRV can be informative during pilot studies and early deployment phases to help identify potential indicators of strain or disengagement not captured by speed or self-report alone. In industrial contexts, such measures are more appropriate for short-term evaluation and redesign iterations (with appropriate privacy safeguards) than for continuous operational monitoring.

#### 5.5.6 Adopt an iterative, human-centered deployment loop

Short pilot cycles that vary collaboration configuration and guidance, and assess performance, perceived safety, workload-related experience, and autonomic activation, can support progressive refinement of feedback timing, modality, and training. This reduces the risk of deploying configurations that appear efficient in the short term but are not sustainable across repeated cycles or prolonged exposure.

Collectively, these guidelines emphasize that successful deployment depends not only on technical capability and safety certification, but also on sustained alignment between system behavior, task demands, and human capabilities in industrial work systems.

### 5.6 Limitations and future work

Several limitations of the present study should be acknowledged.

One limitation concerns the fixed order of feedback conditions across participants. While a consistent sequence was maintained to ensure experimental control, this design choice may introduce potential order effects, including learning, habituation, and fatigue. These factors may have influenced participants’ performance and responses across modalities, and therefore some portion of the observed modality-related effects may remain partially confounded with sequence-related adaptation processes across repeated task exposure. Accordingly, the findings should be interpreted with appropriate caution. In future work, randomized ordering of feedback conditions will be considered in order to further reduce potential order-related effects and to enable direct comparison between randomized and fixed-sequence experimental designs relative to the current findings.

Another limitation is that the participant sample consisted of university students with limited professional experience in industrial environments, which may limit the generalizability of the findings to experienced industrial operators.

Also, the experimental tasks were designed as controlled laboratory abstractions of collaborative work rather than direct replications of industrial production tasks. While this approach enabled controlled comparison of feedback modalities and collaboration configurations, real manufacturing environments involve additional factors such as variability in task complexity, environmental noise, and production pressure.

Future work should extend the present investigation to longitudinal and field-based studies involving professional operators and realistic shop-floor tasks. Such studies could examine learning effects, error and fault management, trust calibration over time, and longer-term physiological regulation during sustained human–robot collaboration.

Beyond the physiological measures considered in this study, additional sensing modalities may further enrich the understanding of user state in human–robot interaction. While HRV provides valuable insights into autonomic regulation, electrodermal activity (EDA) represents a complementary modality with strong sensitivity to rapid changes in arousal and stress. Although EDA was not included in the present study due to its susceptibility to motion artifacts in dynamic interaction scenarios, future work should consider multimodal physiological sensing approaches that combine HRV and EDA. Such integration may enable more robust real-time detection of user state in human–robot collaboration, particularly in field deployment settings.

## 6 Conclusion

This study examined how feedback modalities and collaboration configurations influence operator performance, subjective experience, and physiological responses during human–cobot collaboration. By integrating self-report measures, task performance metrics, and HRV analysis, the work provides an applied perspective on feedback design in collaborative robotic work systems.

The results suggest that feedback modality can shape task outcomes and operator experience within each collaboration scenario, while the observed effects depend strongly on the coordination structure of the task.

In the low-collaboration (turn-taking) task, the absence of feedback enabled faster task completion but was associated with lower perceived interaction quality and descriptively higher autonomic activation. In contrast, multimodal feedback improved perceived comfort and task understanding, albeit with some performance costs.

In the high-collaboration synchronous task, projected assistive interface (PAI) guidance supported the most efficient coordination and yielded the highest levels of perceived comfort and task understanding. At the same time, this condition was associated with higher sympathetic-dominant physiological activation.

These findings suggest a potential trade-off in which richer guidance can enhance coordination efficiency but may also increase cognitive and physiological demands.

These findings further suggest that, within the specific interaction conditions examined in the present study, the examined PAI configuration may support coordination during high-collaboration interaction in addition to providing task-related feedback.

From a design perspective, the findings emphasize that autonomic activation observed during collaborative interaction should not automatically be interpreted as detrimental stress. Instead, physiological responses should be interpreted relative to task demands, interaction transparency, and concurrent performance and subjective outcomes. These interpretations should be considered in light of the statistical results, which indicated that HRV differences across modalities were not statistically significant in Scenario one and only partially significant in Scenario 2.

Under appropriate feedback conditions, elevated activation may reflect task-related engagement that coincided with effective coordination under the specific experimental conditions examined in the present study. Taken together, the findings suggest that collaboration configuration and feedback modality should be co-designed rather than treated as independent system components.

Overall, the findings suggest that collaboration configuration and feedback modality may function as adjustable design parameters, although their effects should be interpreted in relation to the specific task characteristics and experimental context.

Considering these parameters jointly enables the design of collaborative workstations that better balance task efficiency, perceived safety, and sustainable operator performance over time.

By combining subjective and physiological indicators, the present work contributes evidence-informed guidance for designing feedback mechanisms that support effective and sustainable human–cobot collaboration in industrial work systems.

## Data Availability

The original contributions presented in the study are included in the article/Supplementary Material, further inquiries can be directed to the corresponding author.
