# Supplementary material for: Feedback modalities in human-cobot collaboration: experimental evaluation of performance, user experience, and physiological responses
Source: Front Robot AI. 2026 Jul 30;13:1836165. doi: 10.3389/frobt.2026.1836165 (PMC13467822; doi:10.3389/frobt.2026.1836165)
Supplement: Supplementary file 1 [file Supplementaryfile1.pdf]

Supplementary Material

Amir Biton
Yuval Cohen
Shraga Shoval

Associated published article

Feedback modalities in human–cobot collaboration: Experimental evaluation of performance, user experience, and physiological responses

This supplementary document accompanies the published article and provides the supplementary materials referenced throughout the manuscript.

The supplementary material includes the complete post-scenario questionnaire, the Kubios HRV analysis settings, and the additional physiological analyses supporting the reported findings.

Contents

Supplementary Appendix A: Post-Scenario Questionnaire **2**

Supplementary Appendix B: Kubios HRV Analysis Settings **5**

Supplementary Appendix C: Additional Physiological Metrics **6**

Supplementary Appendix A: Post-Scenario Questionnaire

This supplementary appendix provides the complete post-scenario questionnaire administered to participants after completing each experimental scenario. The questionnaire comprises four sections: subjective workload (NASA-TLX), task-specific experience and perceived safety, trust and emotional experience, and general attitudes toward robots (NARS). Participants responded to all items on either a 21-point scale (NASA-TLX) or a 5-point Likert scale. The NARS scale was administered at the end to minimize biasing responses to scenario-specific items. The questionnaire was administered digitally via tablet after completion of each experimental scenario in a quiet and controlled environment. Within each post-scenario questionnaire, participants retrospectively evaluated each feedback modality (i.e., each sub-condition within the scenario) separately using dedicated rating sections for the corresponding interaction experience measures. The appendix presents the overall questionnaire structure and representative item categories used in the study. Some modality-specific outcomes reported in the Results section were derived from individual questionnaire items corresponding directly to specific interaction constructs.

Section 1 – NASA-TLX: Task Load Rating

Participants rated each dimension using a 21-point scale, with anchors at 1 (Very Low) and 21 (Very High). The Raw TLX scoring method was used (i.e., without pairwise weighting). The dimensions were: *Mental Demand*, *Physical Demand*, *Temporal Demand*, *Self-Perceived Performance*, *Effort*, and *Frustration Level*.

Table 1: NASA-TLX dimensions for subjective workload assessment.

**Dimension**	**Rating Scale (1–21)**
Mental Demand	
Physical Demand	
Temporal Demand	
Self-Perceived Performance	
Effort	
Frustration Level	

Section 2 – Task-Specific User Experience and Safety

Please indicate your level of agreement with each statement regarding your experience during the task, using a 5-point Likert scale (1 = Strongly Disagree, 5 = Strongly Agree).

Table 2: Perceived experience and safety while working with the cobot.

**Statement**	**1–5**
I felt safe working alongside the cobot.	
The feedback provided by the cobot was clear and easy to interpret.	
The timing of the cobot's feedback matched well with the task.	
I found the task comfortable to perform.	
I felt in control throughout the task.	
I felt cognitively overloaded during the task. *[reverse-coded]*	
I would trust this cobot in a real work environment.	
I would prefer to perform this task again with the same feedback modality.	

Section 3 – User Trust, Stress, and Emotional Experience

Note: This questionnaire was completed once per scenario, immediately after participants finished all iterations. Within each post-scenario questionnaire, participants retrospectively evaluated each feedback modality (i.e., each sub-condition within the scenario) separately using dedicated rating sections corresponding to the experienced interaction modality.

Please indicate your agreement with the following statements using a 5-point Likert scale (1 = Strongly Disagree, 5 = Strongly Agree).

Table 3: Trust, stress, and emotional experience during the collaborative task.

**Statement**	**1–5**
I trusted the cobot to complete its part of the task accurately.	
I felt that the cobot's actions were predictable.	
I felt that the cobot understood my intentions during the task.	
I experienced moments of stress while collaborating with the cobot.	
I experienced pressure to complete the task faster than I preferred.	
I felt confident in my ability to work with the cobot.	
I would rely on this cobot in a real-world work setting.	
I felt that the cobot adapted to my working pace.	
I felt that the collaboration with the cobot reduced my workload.	
I felt emotionally comfortable throughout the interaction.	

Section 4 – NARS: Attitudes toward Robots

Please indicate how strongly you agree or disagree with the following statements using a 5-point Likert scale (1 = Strongly Disagree, 5 = Strongly Agree).

Table 4: NARS-S1: Negative attitudes toward interaction with robots.

**Statement**	**1–5**
I feel nervous when working near a robot.	
I feel uneasy when robots stare at me.	
I do not trust robots in situations that could be dangerous.	
I feel tense when I have to work with a robot.	
I believe that robots cannot be morally responsible for their actions.	

This section included five items from the Negative Attitudes toward Interaction with Robots subscale (NARS-S1) of the NARS questionnaire. The NARS was administered only post-scenario to assess trait-level attitudes without pre-task priming.

Optional Open-ended Question

Please describe anything in the interaction with the cobot - whether helpful or disruptive - that affected your ability to perform the task effectively:

Supplementary Appendix B: Kubios HRV Analysis Settings

This supplementary appendix presents the Kubios HRV analysis settings applied consistently across all participants and experimental conditions.

All HRV metrics in this study were computed using the **Kubios Premium** software. The analysis followed the standard short-term HRV configuration recommended in the software documentation and relevant literature. The following settings were applied consistently across all participants and experimental conditions:

- **Detrending method:** Smoothness prior's method ($\lambda = 500$) to remove slow nonstationary trends in the RR interval series.
- **Artifact correction:** Automatic, medium sensitivity threshold to replace ectopic beats or missing data segments with cubic spline interpolation.
- **Interpolation rate:** 4 Hz resampling of the RR interval series for spectral analysis.
- **Frequency-domain analysis:**
  - Low Frequency (LF) band: 0.04–0.15 Hz
  - High Frequency (HF) band: 0.15–0.40 Hz
- **Time-domain metrics:** SDNN, RMSSD, pNNxx, mean HR, min HR, and max HR extracted directly from the detrended and corrected RR series.
- **Nonlinear and autonomic balance indices:** SNS and PNS activity indices computed using Kubios' proprietary algorithms based on LF/HF ratio and normalized spectral components.

These settings were selected to ensure reproducibility, alignment with previous HRI and ergonomics research, and comparability with established HRV norms.

Supplementary Appendix C: Additional Physiological Metrics

This supplementary appendix presents the complete set of physiological measures obtained during the experiment.

While the main Results section focuses on the primary HRV indicators (Stress Index, SNS Index, SDNN, and RMSSD), the figures below present the complete set of physiological measures extracted from the Kubios analysis.

These additional metrics include parasympathetic activity indicators (PNS index), frequency-domain measures (LF/HF ratio), and heart rate related measures (maximum heart rate and pNNxx). The inclusion of these metrics provides a comprehensive overview of the autonomic responses observed during the experiment.

Figure 1 presents the complete physiological results for Scenario 1.

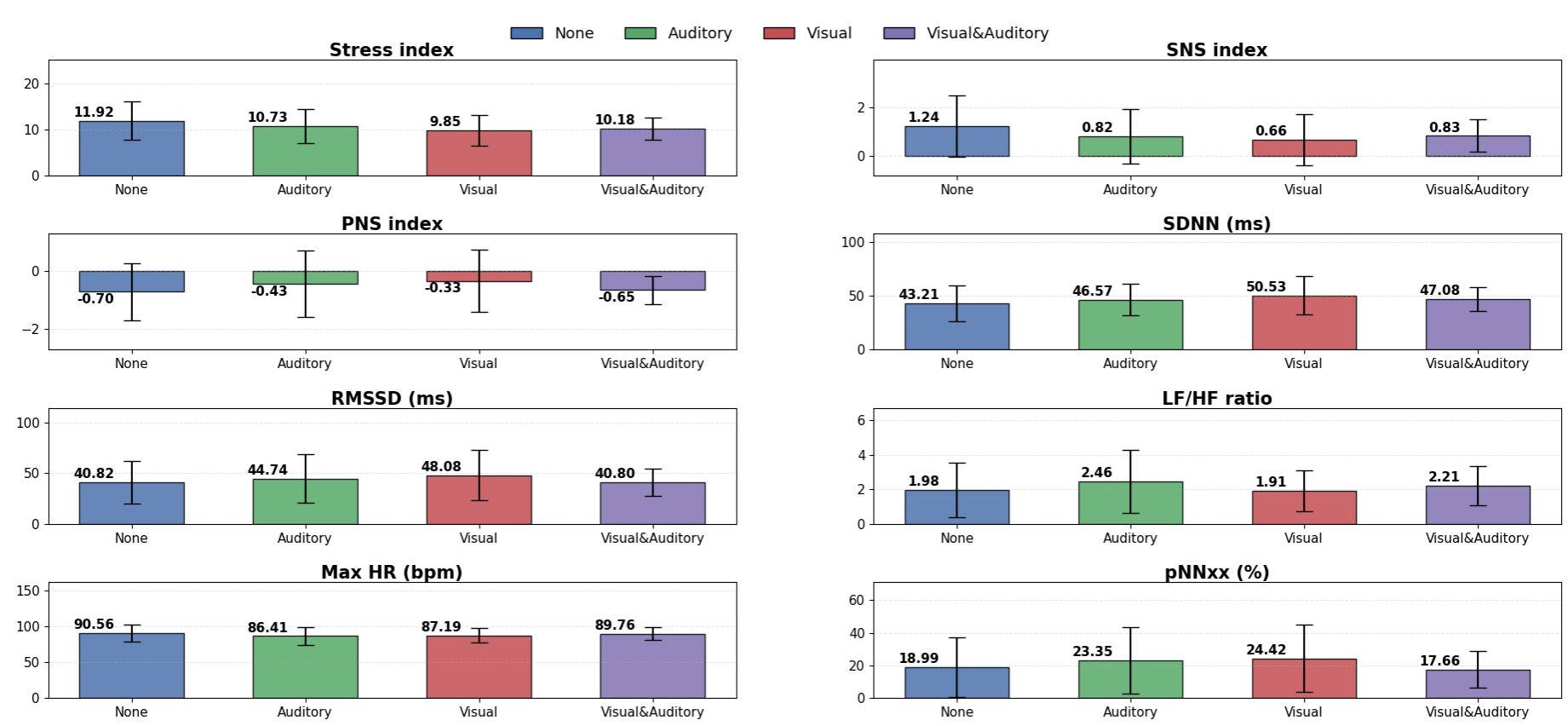

Figure 1: Complete set of physiological measures across feedback modalities in Scenario 1 (low-collaboration turn-taking task). Values represent mean ± SD across participants.

In addition, Figure 2 presents the complete set of physiological measures for Scenario 2 (high-collaboration synchronous task), including all HRV-derived indicators across the five feedback modalities.

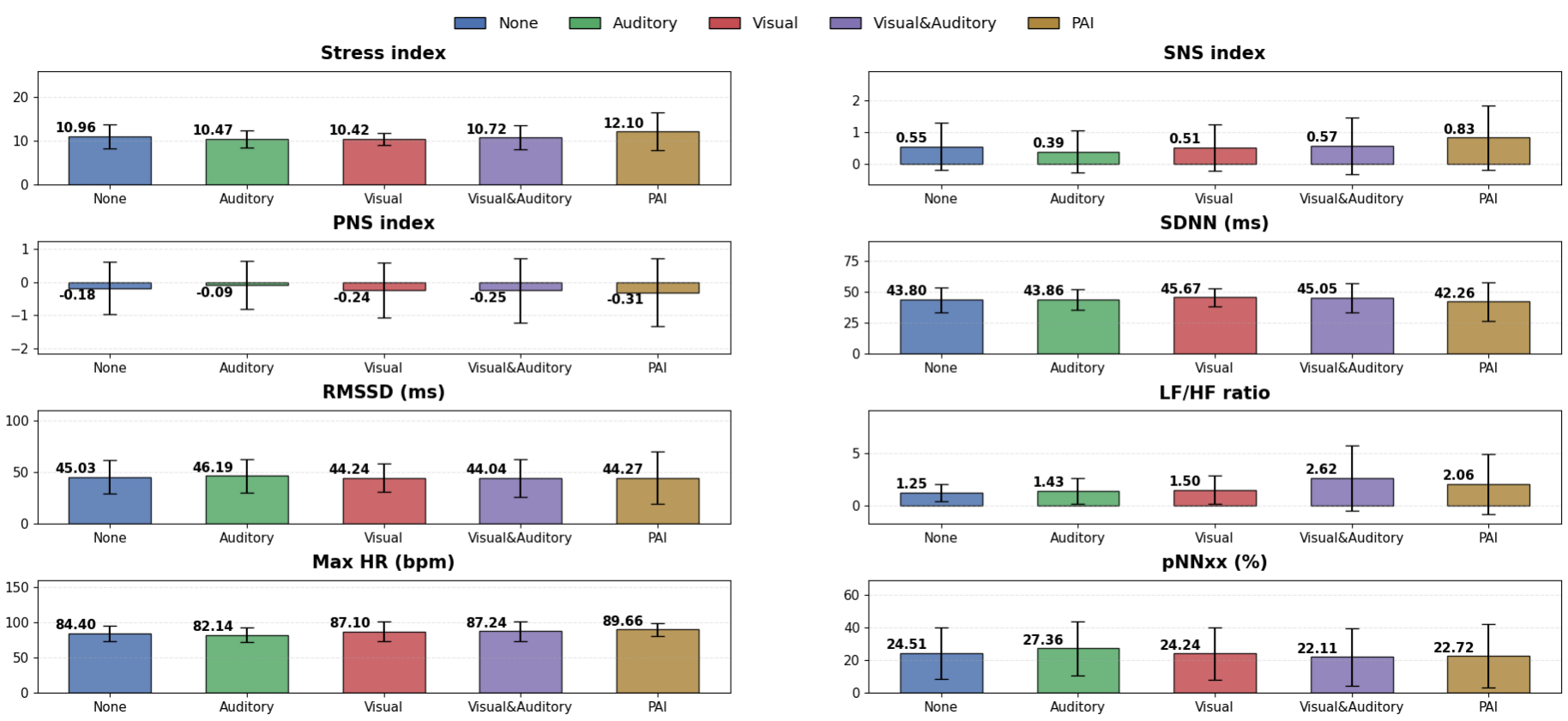

Figure 2: Complete set of physiological measures across feedback modalities in Scenario 2 (high-collaboration synchronous coordination task). Values represent mean ± SD across participants.
